# Characterization of Gene Families Encoding Beta-Lactamases of Gram-Negative Rods Isolated from Ready-to-Eat Vegetables in Mexico City

**DOI:** 10.3390/ht7040036

**Published:** 2018-11-23

**Authors:** Rosalino Vázquez-López, Sandra Solano-Gálvez, Bertha A. León-Chávez, María R. Thompson-Bonilla, Tayde Guerrero-González, Eduardo Gómez-Conde, Daniel Martínez-Fong, Juan A. González-Barrios

**Affiliations:** 1 CICSA Facultad de Ciencias de la Salud Universidad Anáhuac Mexico Campus Norte, Huixquilucan, Estado de Mexico 52786, Mexico; vazquezrosalino@yahoo.com; 2 Departamento de Microbiología y Parasitología, Facultad de Medicina, Universidad Nacional Autónoma de Mexico. Coyoacán, Ciudad de Mexico 04510, Mexico; solano-sandra@hotmail.com; 3 Laboratorio de Investigaciones Químico-Clínicas, Facultad de Ciencias Químicas, Benemerita Universidad Autónoma de Puebla, San Manuel, Ciudad Universitaria, Puebla 72570, Mexico; alileonch@gmail.com; 4 Laboratorio de Medicina Genómica, Hospital Regional “Primero de Octubre”, ISSSTE, Av. Instituto Politécnico Nacional 1669, Lindavista, Gustavo A. Madero, Ciudad de Mexico 07300, Mexico; rociothompson@yahoo.com.mx (M.R.T.-B.); taygercita.19@gmail.com (T.G.-G.); 5 División de Investigación en Salud, Unidad Médica de Alta Especialidad (UMAE), Hospital de Especialidades, Centro Médico Nacional General de División “Manuel Ávila Camacho”, Instituto Mexicano del Seguro Social (IMSS), Puebla 72090, Mexico; eduardo.gomezc@imss.gob.mx; 6 Departamento de Fisiología Biofísica y Neurociencias, Centro de Investigación y Estudios Avanzados, Av. Instituto Politécnico Nacional 2508, San Pedro Zacatenco, Gustavo A. Madero, Ciudad de Mexico 07360, Mexico; martinez.fong@gmail.com

**Keywords:** Enterobacteriaceae, antibiotics, beta-lactamases, beta-lactam resistome, whole genome sequencing

## Abstract

Beta-lactam resistant bacteria, which are commonly resident in tertiary hospitals, have emerged as a worldwide health problem because of ready-to-eat vegetable intake. We aimed to characterize the genes that provide resistance to beta-lactam antibiotics in Enterobacteriaceae, isolated from five commercial salad brands for human consumption in Mexico City. In total, twenty-five samples were collected, grown in blood agar plates, and the bacteria were biochemistry identified and antimicrobial susceptibility testing was done. The carried family genes were identified by endpoint PCR and the specific genes were confirmed with whole genome sequencing (WGS) by Next Generation Sequencing (NGS). Twelve positive cultures were identified and their microbiological distribution was as follows: 8.3% for *Enterobacter aerogene* (*n* = 1), 8.3% for *Serratia fonticola* (*n* = 1), 16.7% for *Serratia marcesens* (*n* = 2), 16.7% for *Klebsiella pneumoniae* (*n* = 2), and 50% (*n* = 6) for *Enterobacter cloacae*. The endpoint PCR results showed 11 colonies positive for *bla*BIL (91.7%), 11 for *bla*SHV (91.7%), 11 for *bla*CTX (97.7%), 12 for *bla*DHA (100%), four for *bla*VIM (33.3%), two for *bla*OXA (16.7%), two for *bla*IMP (16.7%), one for *bla*KPC (8.3%), and one for *bla*TEM (8.3%) gen; all samples were negative for *bla*ROB, *bla*CMY, *bla*P, *bla*CFX and *bla*LAP gene. The sequencing analysis revealed a specific genotype for *Enterobacter cloacae* (blaSHV-12, blaCTX-M-15, blaDHA-1, blaKPC-2); *Serratia marcescens* (blaSHV-1, blaCTX-M-3, blaDHA-1, blaVIM-2); *Klebsiella pneumoniae* (blaSHV-12, blaCTX-M-15, blaDHA-1); *Serratia fonticola* (blaSHV-12, blaVIM-1, blaDHA-1); and, *Enterobacter aerogene* (blaSHV-1, blaCTX-M-1, blaDHA-1, blaVIM-2, blaOXA-9). Our results indicate that beta-lactam-resistant bacteria have acquired integrons with a different number of genes that provide pan-resistance to beta-lactam antibiotics, including penicillins, oxacillins, cefalosporins, monobactams, carbapenems, and imipenems.

## 1. Introduction

The intake of ready-to-eat salad greens from commercial sources is a worldwide health practice that might be a potential cause of gastrointestinal (GI) disease. Because of traditional agricultural techniques, fresh foods, such as fruits and vegetables, are usually contaminated with a great deal of diverse bacteria [1]. Frequently, these foods are eaten raw or without an appropriate sanitization process [2]. Food safety comprises the conditions and practices that preserve the quality and purity of food in order to prevent illness by its intake [3]. The intake of food and water with poor hygiene quality can cause GI illnesses [4]. It is estimated that around 2195 children die every day because of infectious diarrhea, with a mortality rate greater than that associated with AIDS, malaria, and measles together [5]. GI infections are the second cause of death in children younger than five years-old worldwide [5]. This situation becomes even more critical when dealing with multiresistant *Enterobactrias* [6]. Beta-lactam antibiotics are widely used as an election treatment for GI infections in pediatric patients. However, the increasing number of beta-lactam-resistant Enterobacteriaceae in the last two decades has dramatically decreased the availability of effective antibiotics [7].

The enzymatic degradation of beta-lactam rings by beta-lactamases is the most effective resistance mechanism that bacteria have for fighting back against the antibacterial effect of beta-lactam antibiotics [8]. The genes encoding the beta-lactamases have been spread widely between different bacterial populations, which are mainly due to their mobile genetic elements, such as carrier plasmids and transposons. The organization of integrons, carriers of multiple gene-encoding beta-lactamases that form multi-drug resistance cassettes, represents another mechanism that contributes to the increase of positive bacterial populations to the beta-lactamases [9]. Beta-lactamases (*bla*) are classified depending on their structure or resemblance in the amino acid sequence (Classes A-D) [10] or according to their function (Groups I-IV) [11]. There are two types of beta-lactamases: serine-beta-lactamases and metallo-beta-lactamases [12]. Classes A, C, and D are serin-beta-lactamases. All members of these groups share the same mechanism of action and have a characteristic serine in the active site. This serine is deprotonated to produce a nucleophile serine that can induce a nucleophilic attack in the beta-lactam ring, generating an acyl-enzyme intermediate which is easily hydrolyzed on a general basis [9]. The members of class B are metallo-beta-lactamases. These enzymes perform a zinc-dependent nucleophilic attack [9,12]. The class A or group II enzymes hydrolyze penicillin and cephalosporin. Several gene families of these beta-lactamases have been identified, such as *bla*TEM, *bla*SHV, *bla*CTX, *bla*CFX, *bla*LAP, *bla*ROB, and *bla*KPC [13]; however, some bacteria have shown carbapenem resistance [14]. The members of class B of beta-lactamases hydrolyze carbapenems and the genes encoding them are *bla*IMP (Imipenem-resistant) and *bla*VIM (Verona Integron-encoded Metallo type beta-lactamases) [15]. Class C beta-lactamases belonging to group I have cefalosporinase activity. AmpC beta-lactamase [16] was the first isolated enzyme from this class. Later, isolations were named according to the antibiotic in which they showed inhibitory action. For example, *bla*CMY has activity against cephamycin [17], *bla*_FOX_ against cefoxitin, *bla*MOX against moxalactam, and *bla*LAT against latamoxef. Other C group beta-lactamases were named according to the place of isolation, such as *bla*MIR-1 (Miriam Hospital in Providence, R.I.) or *bla*DHA (Dhahran hospital in Saudi Arabia). The C *bla*BIL beta-lactamase owes its name to the patient from which it was isolated (Bilal) [18]. Class D beta-lactamases have oxacillinase activity and they are encoded by *bla*_OXA_ gene [19]. A worldwide increase of bacterial strains having *bla*_OXA_ with weak resistance to carbapenems has been reported [14,20]. The increase of bacterial strains resistant to beta-lactams has been reported not only in clinical isolations, but also from diverse food sources, including vegetables [21,22]. 

Recently, the Mexican health system has been promoting the intake of vegetables, fruits, and herbs to prevent and reduce obesity, which is a health problem in Mexico City [23]. This lofty promotion entails another health risk, because the great majority of those products is grown by traditional agriculture techniques in Mexico and they have been related to the high prevalence of GI disease [24]. Therefore, this work aimed to identify the gene families coding for beta-lactamases in Enterobacteriaceae isolated from vegetable samples from salads sold in Mexico City, labelled as suitable for human consumption. Our results show the high probability of the contamination of fresh vegetables with pathogenic multi-resistant Enterobacteriaceae and they provide insight into the beta-lactam pan-resistance mechanism of Enterobacteriaceae.

## 2. Material and Methods 

### 2.1. Bioinformatics Analysis and Primer Design

We obtained the beta-lactamase integrons and DNA sequences from the National Center for Biotechnology Information. We used all the sequences of beta-lactamase families reported in order to determine the conserved sequence in each gene family encoding beta-lactamases. The integrons and DNA alignments were made with ClustalW software (V2.1, Conway Institute UCD, Dublin, Ireland) [25] according to DNA loss model parameters using a gap of 0.05 [26,27]. The phylogenetic trees were edited with the FigTree software (V1.4.2 Institute of Evolutionary Biology, Ashworth Laboratories, Kings Buldingg, Edimburgh, UK), and the phylogenetic analysis was made according to the procedure that was described by Martinez-Perez et al., 2009 [28]. We used the conserved sequence identified in the alignment for all gene to design specific and degenerate primers by using PerPrimer Software (V1.1.21, Owen J. Marshall, Melbourne, Australia) [29] following the next criteria, length (18–25 bp), Tm (60–62 °C), GC (40–60%), ∆T° (1 °C), Amplicons (83–230 bp) to run all the endpoint PCRs reactions in the same plate.

### 2.2. Sampling

Under sterile conditions, samples of 2 cm^2^ were collected from the vegetables of five different salad brands, labelled as suitable for human consumption and sold in marketplaces in Mexico City. The samples were cultured in BHI (blood and heart infusion; Becton Dickinson; Franklin Lakes, NJ, USA) incubated at 37 °C for 24 h.

### 2.3. Bacterial Isolation

After its incubation, 10 μL from the BHI were taken and subcultured in MacConkey agar (Becton Dickinson; Franklin Lakes, NJ, USA), and then incubated at 37 °C for 24 h. The colonies obtained were subcultured in blood agar and incubated at 37 °C for 24 h.

### 2.4. Bacterial Identification

The bacterial identification was made from the pure colonies that were obtained from blood agar using Gram staining and oxidase, Indol and biochemistry tests. The BD BBL Gram Stain Kit (Becton Dickinson; Franklin Lakes, NJ, USA) was used. Oxidase and Indol tests were carried out by using BBL DrySlide Oxidase kit (Becton Dickinson, Franklin Lakes, NJ, USA) and BBL DrySlide Indole kit (Becton Dickinson; Franklin Lakes, NJ, USA) according to the manufacturer’s specifications. The bacterial identification was performed using the BBL Crystal Enteric/Nonfermenter ID system (Becton Dickinson; Franklin Lakes, NJ, USA). Briefly, bacterial suspensions were made depositing two or three medium-sized colonies (2 to 3 mm) in BBL Crystal Inoculum Broth (Becton Dickinson; Franklin Lakes, NJ, USA). That inoculum was adjusted to a Mac Farland 1.0 scale (Expected CFU/mL 3.0 × 10^8^). To adjust the scale, a CrystalSpec Nephelometer (Becton Dickinson; Franklin Lakes, NJ, USA) was used. The inoculum was deposited in BBL Crystal Enteric/Nonfermenter ID Kit plates, incubated at 37 °C for 18 h, without CO_2_ and 40 to 60% humidity. Finally, the plates were read using a BBL Crystal AutoReader (Becton Dickinson; Franklin Lakes, NJ, USA) and the results were analyzed with the BBL Crystal MIND Software (Becton Dickinson; Franklin Lakes, NJ, USA).

### 2.5. Antimicrobial Susceptibility Testing

Antimicrobial susceptibility testing was carried out by the Kirby–Bauer method under the Clinical and Laboratory Standards Institute protocol (NCCLS standards). The pure colonies obtained from blood agar were resuspended in bacterial suspensions were made depositing two or three medium-sized colonies (2 to 3 mm) in BBL Crystal Inoculum Broth (Becton Dickinson; Franklin Lakes, NJ, USA). The obtained inoculum was adjusted while using a Mac Farland 0.5 reading (Expected CFU/mL 1.5 × 10^8^), and cultured in Müeller Hinton 150 × 15 mm^2^ media BD BBL (Becton Dickinson Franklin Lakes, NJ, USA). The antibiotic discs were applied with the Sensi-Disc Designer Dispenser System. The antibiotics panel was conformed by ampicillin (10 µg), ampicillin/sulbactam (10/10 µg), mezlocillin (75 µg), carbenicillin (100 µg), piperacillin/tazobactam (100/10 µg), cefazolin (30 µg), cefaclor (30 µg), cefepime (30 µg), cefoperazone (75 µg), and cefotetan (30 µg) from Becton Dickinson (Franklin Lakes, NJ, USA)

### 2.6. DNA Extraction

#### 2.6.1. Crude Extract

Aliquots of 5 mL of Luria–Bertani broth (LB) were inoculated with the isolated bacteria and incubated overnight at 37 °C with continuous shaking (200 rpm). The bacterial culture was centrifuged at 4000 rpm for five minutes at room temperature (RT). The bacterial pellet was resuspended in 1 mL of RNase and DNase-free deionized water and then heated at 94 °C for 10 min, followed by thermal shock in ice. Finally, the sample of lysed bacterial was kept at −80 °C until use.

#### 2.6.2. Genomic DNA Extraction

Samples of 500 µL of overnight bacterial culture was subjected to genomic DNA isolation by using an RTP pathogen kit (Invitek, Berlin, Germany) following the manufacturer’s instructions. Briefly, a bacterial pellet was collected by centrifugation at 13,000 rpm for 5 min, suspended with 400 µL of resuspension buffer, and placed into an extraction L tube to be incubated at 37 °C for 10 min and switched to 95 °C for 10 min. Then, 400 µL of binding buffer were added and loaded onto the RTA spin filter set, incubated for one minute, and centrifuged at 9300× *g* for one minute, then the column was replaced into a new collector tube, loaded with 500 µL of wash R1 buffer, and centrifuged at 9300× *g* for a min. Again, the column was replaced into a new collector tube, loaded with 700 µL of wash R2 buffer and centrifuged at 9300× *g* for one minute; then, the column was replaced into a new collector tube and centrifuged at 12,000× *g* for 4 min. Finally, the column was replaced into a new 1.5 mL Eppendorf tube and 60 µL of elution buffer prewarmed to 80 °C was added, incubated for 3 min at RT, and centrifuged at 9300× *g* for 2 min. The eluted DNA solution was quantified by absorbance and its integrity was verified by 2%-agarose gel electrophoresis. The sample was kept at −20 °C until use.

### 2.7. Plasmid DNA Extraction

Samples of overnight bacterial culture (100 mL) were subjected to a plasmid DNA isolation by using PureLink HiPure Plasmid Midiprep Kit (Invitrogen; Carlsbad, CA, USA) following the manufacturer’s instructions. Briefly, the bacterial culture was harvested by centrifugation at 4000× *g* for 10 min, then 10 mL of resuspension buffer (R3) and 10 mL of lysis buffer (L7) were added to the bacterial pellet, and incubated at RT for 5 min. Then, 10 mL of precipitation buffer (N3) was added and centrifuged at 12,000× *g* for 10 min at RT. The supernatant was placed onto a pre-equilibrated maxi-column using 30 mL equilibrium buffer (EQ1) and eluted by gravity. Then, 60 mL of wash buffer (W8) was added, eluted by gravidity, and the eluent was discarded. Finally, 15 mL of elution buffer (E4) was added, eluted by gravity, and the eluent was added with 10.5 mL of isopropanol and centrifuged at 12,000× *g* for 30 min at 4 °C. The DNA pellet was washed with 5 mL of 70% ethanol and centrifuged at 12,000× *g* for 5 min at 4 °C. The DNA pellet was dried at RT for 10 min and finally resuspended in 250 µL of sterile RNase- and DNase-free water. The Plasmid DNA concentration was quantified by digital spectrophotometry (NanoDrop 8000, Thermo Fisher Scientific; Wilmington, DE, USA) and its integrity was verified by electrophoresis in 2% agarose gel, and the plasmid DNA sample was kept at −20 °C until use.

### 2.8. Endpoint PCR

The endpoint PCR to identify the β-Lactamase gene family was made using the primers sense and antisense described in Table 1. Amplification was made in 25 μL of the reaction mixture containing 2.5 μL of 10× PCR buffer (100 mM TRIS·HCI, 15 mM MgCl_2_, and 500 mM KCl, pH 8.3), 200 nM each dNTP, 10 μM each primer, 1 U Taq DNA polymerase (Invitrogen; Carlsbad, CA, USA), and 2 μL of crude extract or 10 ng of DNA (genomic or plasmid). The PCR conditions were 94 °C for 5 min, then 35 cycles of 94 °C for 30 s, 60 °C for 30 s, 72 °C for 30 s, and finally 72 °C for 10 min. The PCR products were analyzed in 2% agarose gel electrophoresis and the image was digitized in a Gel Logistic 2200 Digital Imaging System (Carestream, New Haven, CT, USA).

### 2.9. Whole Genome Sequencing (WGS) and Genotype Analysis

The WGS was performed from indexed libraries that were prepared using a standard Illumina Nextera XT DNA Sample Preparation Kit (FC-131-1096) for small genomes and was sequenced on the MiSeq platform (Illumina; San Diego, CA, USA). Adapters and barcodes were trimmed by the default setting in the Illumina experiment manager, generating 300 bp paired-end reads. The quality of the unprocessed reads was assessed using FastQC High Throughput Sequence QC Report v:0.11.5 (Babraham Bioinformatics, Babraham Institute; Cambridge, UK) [30]. A minimum Q score of more than 30 for at least 85% of all reads was attained. All reads were mapped using BWA-MEM aligner version 0.7.7-r441 (Wellome trust, Sanger Institute, Hinxton, UK) [31] and SAMtools version 1.3.1 (Wellome trust, Sanger Institute, Hinxton, UK). The NOVO genome assembly was done using the SPAdes Genome Assembler software version 3.11 (CAB, St. Petesburg State University, Russia) [32].

The metagenomic analysis for the taxonomic classification of bacteria was done by using the software Kraken taxonomic sequence classification system Version 0.10.5-beta (CCB, Johns Hopkins University, Baltimore, MD, USA) [33]. The beta-lactamase genes were identified by the comparative analysis while using the Basic Local Alignment Search Tool (BLAST, NCBI-NIH, Bethesda, MD, USA) [34].

## 3. Results

The bioinformatics analysis included 1007 complete sequences of different genes encoding beta-lactamases reported in the Genbank, of which 936 sequences were used for primer design after discarding 71 repeated sequences (Table 1).

**Figure 1:**
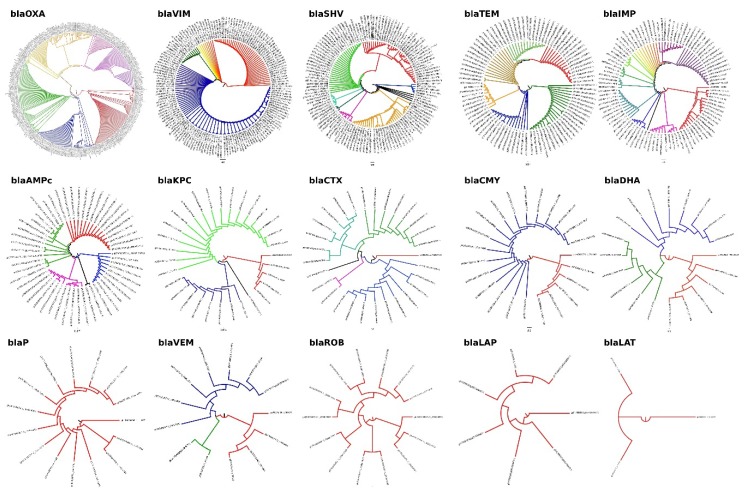
Phylogenetic analysis of the gene family that confer beta-lactam resistance: all sequences were downloaded from NCBI GeneBankt and aligned in ClustalW. The phylogenetic trees were edited in FigTree v 1.4.0. All software was running in a Debian (Stretch) machine.

From the aligned sequences, we constructed fourteen phylogenetic (Figure 1), which allowed the identification of the more conserved regions for each family of beta-lactamase genes. These conserved sequences were used to design all primers that were used in the endpoint PCR reactions (Table 2) in order to identify the gene families of beta-lactamases that were carried by the contaminated bacteria.

**Figure 2:**
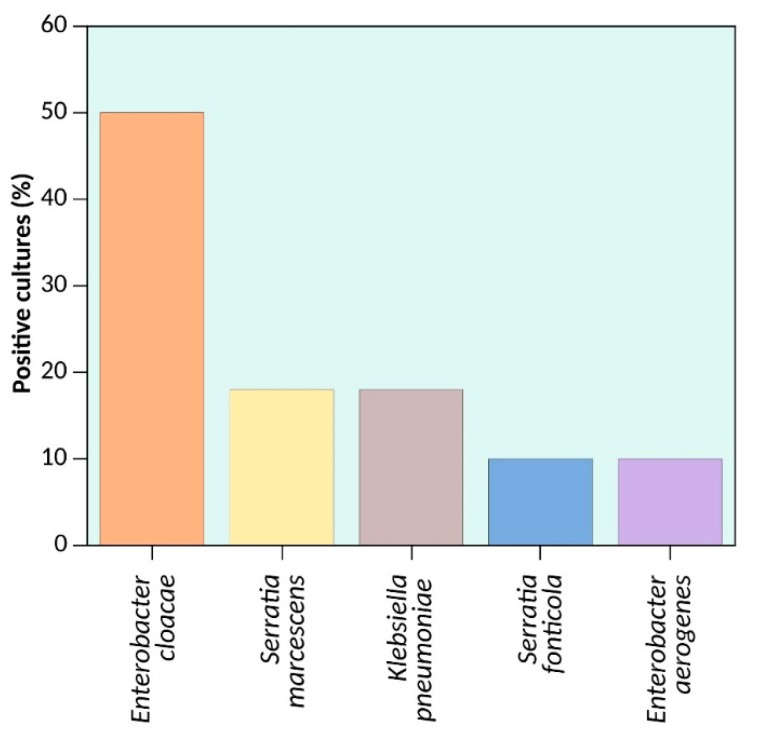
Bacterial frequency. The graph shows the percentage of different bacteria species isolated from sample ready-to-eat salad greens sold in supermarkets placed in Mexico City.

From the sampling of five different salad brands sold in Mexico City and labelled as suitable for human consumption, 12 different Enterobacteriaceae strains from five bacterial species were isolated. The distribution of the identified bacteria was 8.3% of *Enterobacter aerogenes* (*n* = 1), 8.3% of *Serratia fonticola* (*n* = 1), 16.7% of *Serratia marcescens* (*n* = 2), 16.7% *Klebsiella pneumoniae* (*n* = 2), and 50% (*n* = 6) of the samples were identified as *Enterobacter cloacae* (Figure 2, Table 3). All of these data were confirmed by whole genome sequencing using NGS (Table 4) 

In the antimicrobial susceptibility testing, 100% of the isolated Enterobacteriaceae strains showed resistance to ampicillin and carbenicillin, and 8.3% (*n* = 1) showed resistance to mezlocillin. The antibiotic resistance towards cephalosporins was as follows: 75% (*n* = 9) were resistant to cefazolin, 67% (*n* = 8) to cefaclor, 8.3% (*n* = 1) to cefotetan and 8.3% (*n* = 1) to cefoperazone showing medium resistance, and 100% were sensitive to cefepime. As to the beta-lactam and beta-lactamase inhibitor combination (sulbactam or tazobactam), 33% (*n* = 4) showed resistance and 33% (*n* = 4) medium resistance to ampicillin + sulbactam, and 100% were sensitive to piperacillin + tazobactam. 

**Figure 3:**
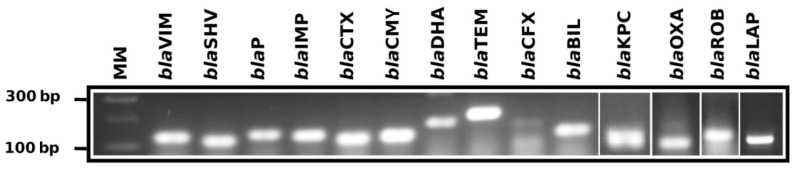
End point polymerase chain reaction for the identification of beta-lactamase gene families. 3% agarosa gel electrophoresis showing the positives amplicons for gene families encoding beta-lactamase, MW = marker (100 bp), *bla*VIM (133 bp), *bla*SHV (106 and 141), *bla*P (204 bp), *bla*IMP (183 and 192 bp), *bla*CTX (116, 83 or 226 bp), *bla*CMY (123 or 160 bp), *bla*DHA (200, 147 or 117), *bla*TEM (213), *bla*CFX (177 bp), *bla*BIL (128 bp), *bla*KPC (117 bp), *bla*OXA (114 bp), *bla*ROB (126 bp), and *bla*LAP (126 bp).

The end point PCR was able to identify the gene families with the phenotype that was responsible for beta-lactamase resistance (Figure 3) and showed that 91.7% (*n* = 11) of characterized Endobacteriaceae strains were positive to *bla*BIL, 91.7% (*n* = 11) to *bla*SHV, 83.3% (*n* = 10) to *bla*CTX, 75% (*n* = 9) for *bla*DHA, 33.3% (*n* = 4) to *bla*VIM, 16.7% (*n* = 2) to *bla*OXA, 16.7% (*n* = 2) to *bla*IMP, 8.3% (*n* = 1) to *bla*KPC and 8.3% (*n* = 1) to *bla*TEM (Table 4). All identified strains were negative for gene families to encode *bla*ROB, *bla*CMY, *bla*P, *bla*CFX and *bla*LAP; these data were also confirmed by whole genome sequencing of isolated strains (Table 5 and Table 6). 

The specific gene expression pattern was analyzed, identifying the following genotypes: *Enterobacter cloacae* (*bla*SHV-12, *bla*CTX-M-15, *bla*DHA-1, *bla*KPC-2); *Serratia marcescens* (*bla*SHV-1, *bla*CTX-M-3, *bla*DHA-1, *bla*VIM-2); *Klebsiella pneumoniae* (*bla*SHV-12, *bla*CTX-M-15, *bla*DHA-1); *Serratia fonticola* (*bla*SHV-12, *bla*VIM-1, *bla*DHA-1) and *Enterobacter aerogene* (*bla*SHV-1, *bla*CTX-M-1, *bla*DHA-1, *bla*VIM-2, *bla*OXA-9). However, in the NGS analysis, all of the bacterial strains tested were negative to blaBIL (Table 7).

The strains that were isolated from ready-to-eat vegetable salads showed a broad pattern of resistance to beta-lactam antibiotic. The analysis of the different families of gene responsible for this resistance pattern showed a mixed genotype of multiple gene families encoding beta-lactamase and were specific for each strain, conformed from three (*bla*VIM + *bla*SHV + *bla*BIL) to six (*bla*OXA + *bla*VIM + *bla*SHV + *bla*CTX + *bla*DHA + *bla*BIL) gene families of beta-lactamases (Table 4).

## 4. Discussion

The implementation of food safety measures has been shown to have a great cost–benefit impact in the developing world, decreasing healthcare costs by $25.5 USD for each $1.0 USD invested in hygiene measures [35]. To contribute to this condition, both government organisms and some food industry enterprises have implemented new technologies that are dedicated to providing products with a high quality and safety for human consumption. An example is the non-traditional agriculture techniques for growing hydroponic vegetables suitable for immediate consumption by humans as salads. Some studies have revealed that human beings are exposed to a wide range of bacterial species due to the consumption of fresh fruits and raw vegetables. The diversity of contaminant organisms has been shown to depend on the vegetables consumed and the kind of agricultural technique used for the food production [36]. There are reports of multi-resistant Enterobacteriaceae (evaluated by the Kirby–Bauer method) isolated from vegetables for human consumption [37]. This is why the intake of raw green salads frequently leads to GI disease outbreaks, especially in developing countries [37].

One molecular mechanism by which bacteria acquire resistance is the production of beta-lactamase enzymes that annihilate the bactericidal effect of beta-lactam drugs. Some Enterobacteriaceae, which are multi-resistant to a wide range of beta-lactam drugs that produce extended-spectrum beta-lactamases, classified as ESBL-producing Enterobacteriaceae, represent a major threat to human health. Usually, ESBL-producing Enterobacteriaceae are resistant to a wide variety of penicillins and cephalosporins, including broad-spectrum cephalosporins. The USA Health Agency reported 140,000 annual infections caused by Enterobacteriaceae during 2013, and 18% of these cases were resistant to beta-lactams that are associated to ESBL enzyme production, 12% of Enterobacteriaceae infections were caused by Klebsiella, and 6% by Escherichia coli, both of which are ESBL-producing. It has been estimated that multi-resistant ESBL-producing Enterobacteriaceae cause 1700 annual deaths in the USA. Patients with bacteremia caused by ESBL-producing Enterobacteriaceae have a rate of mortality 57% higher than that of patients with bacteremia caused by non-resistant Enterobacteriaceae [38,39]. Due to the severity of ESBL-producing Enterobacteriaceae infections and to the limited therapeutic options, the indicated medical treatment consists of the use of beta-lactam antibiotics from the Carbapenem family. However, recent reports have shown that Carbapenemase-producing Enterobacteriaceae (CPE) have spread all over the world during the last years—a situation that complicates their treatment. It has been estimated that 9300 infection cases are caused by carbapenem-resistant Enterobacteriaceae (CRE) each year, of which 7900 are associated to CRE–Klebsiella and 1400 to CRE–Escherichia coli. These infections are responsible for more than 600 deaths annually [36,37,38,39].

In this study, we isolated Enterobacteriaceae with high pathogenic potential from ready-to-eat vegetables that are sold in supermarkets located in Mexico City. The isolated Enterobacteriaceae were *Enterobacter cloacae, Enterobacter aerogenes, Serratia fonticola, Serratia marcescens*, and *Klebsiella pneumoniae*. All of the organisms isolated showed multi-resistance to beta-lactam antibiotics, tested by the Kirby–Bauer method. The antibiogram results showed resistance to penicillin and cephalosporin. Ampicillin is considered to be a broad-spectrum penicillin due to its use in gastrointestinal infections for some Enterobacteriaceae [40]. However, our study showed that all Enterobacteriaceae isolated were resistant to ampicillin. Carbenicillin is also an antibiotic in the treatment of Enterobacteriaceae infections, specifically against *Pseudmonas* spp. [41]. Our study also showed that the Enterobacteriaceae isolated were resistant to carbenicillin. The mezlocillin is mainly used against both *Pseudomonas* spp. and *Klebsiella* spp. [42] and, with the exception of one strain, all the Enterobacteriaceae isolated were sensitive to this antibiotic. In addition, 75% and 67% of all Enterobacteriaceae isolated were resistant to the antibiotic effect of cefazolin and cefaclor, respectively, and 99% showed medium sensitivity. All Enterobacteriaceae isolated were resistant to cefoperazone. The sensitivity to cefepime was not tested. Finally, 66% of isolated Enterobacteriaceae showed different degrees of resistance to ampicillin and beta-lactamase inhibitor combination, while 33% were resistant to sulbactam and 33% showed medium sensitivity. These data show—by the Kirby–Bauer method—that the isolated Enterobacteriaceae have a multi-resistance pattern to beta-lactam antibiotics, including penicillins and cephalosporins, and even were resistant to beta-lactamase inhibitors. These results are in agreement with work done on nitrogen-fixing legumes, where the pattern of multi-resistance was evaluated by a disk diffusion method for β-lactam antibiotics, with reported resistance to Amoxicillin, Ampicillin, Cefadroxil, Ceftriaxone, Oxacillin, and Vancomycin [43]. The food contamination of ready-to-eat salads distributed freely in Mexico City has the potential to become a trigger vector of an epidemic of gastrointestinal infection that is caused by multi-resistant Enterobacteriaceae, which could compromise the lives of patients at risk by their difficult treatment.

Our results with bacterial lysates or plasmid coincided with the identification of the gene families encoding beta-lactamases that are involved in the multi-resistance pattern to beta-lactam antibiotics. We demonstrated that 92% of all Enterobacteriaceae isolated carry at least one member of the beta-lactamase gene family that is associated with cephalosporin resistance, and that 100% were positive to the *bla*DHA gene family, which is responsible for cephalosporin resistance [44]. In addition, 91.7% of the Enterobacteriaceae isolated were positive to the *bla*SHV gene family associated with penicillin and cephalosporin resistance, also related to two grand phenomena of resistance: first, to ESBL-producing Enterobacteriaceae and resistance to beta-lactamase inhibitors [8,45,46]. The spectrum of ESBL-producing Enterobacteriaceae includes penicillin, broad-spectrum cephalosporins, and monobactams [46]. We demonstrated that 16.3% of the Enterobacteriaceae isolated carry a *bla*CTX gene family member, causing resistance to penicillin and broad spectrum cephalosporins, and this also was associated to ESBL-producing Enterobacteriaceae. We found that 8.3% of all strains carry the *bla*TEM gene family; in previous works, associates to ESBL-producing Enterobacteriaceae have been found [8]. The *bla*VIM gene family was present in 33.3% of Enterobacteriaceae isolated, *bla*IMP were in 16.7%, *bla*OXA in 16.7%, and *bla*KPC were positives in 8.3%, all of which have been associated with resistance to carbapenem [41,47,48]. These results indicate that the beta-lactam-resistant Enterobacteriaceae that were isolated from our samples of ready-to-eat vegetables have accumulated integrons with a different number of genes that confer a great variety of beta-lactam antibiotic resistance, including penicillins, oxacillins, cephalosporins, monobactams, carbapenems, and imipenems. However, the presence of a single family of gene-encoding beta-lactamase does not explain by itself the pan-resistance to beta-lactams. This is why the analysis of genotype was crucial to explain this phenomenon. In this study, the beta-lactam resistome drugs identified at least one member of the gene families encoding beta-lactam drugs. The resistome evidenced in different gram-negative Enterobacteriaceae isolated from ready-to-eat vegetables is integrated at least by one extended-spectrum beta-lactamase in combination with a specific beta-lactamase as cephalosporinases, cefotaximases, imipenemases, oxacillinases or carbapenemases. This paper reports the presence of beta-lactam resistome in ready-to-eat salads, sold in supermarkets in Mexico City, contaminated with carrier enterobacteria of resistance genes to last line beta-lactam antibiotics exclusively used in hospital patients.

## 5. Conclusions

All of these results together demonstrate that ready-to-eat vegetables for human intake in Mexico City are contaminated with potentially pathogenic multi-resistant Enterobacteriaceae; their intake might lead to GI diseases with a high difficulty of treatment. Therefore, food processing under strict safety measures and quality standards must be followed by food industries and supervised by the Health National Agency in order to avoid GI disease outbreaks. In addition, our results provide insight into the beta-lactam pan-resistance mechanism of Enterobacteriaceae. Our methodological approach can be implemented by food enterprises as a routine safety control of food processing.

## Figures and Tables

**Table 1:** Gene families encoding beta-lactamases used in the phylogenetic analysis and primers design.

*Gen Family*	*Sequences*	*Discarded*	*Analyzed*	*Discarded (%)*	*Analyzed (%)*
*bla*OXA	322	9	313	2.80	97.20
*bla*VIM	186	29	157	13.24	71.68
*bla*SHV	129	3	126	2.32	97.67
*bla*TEM	102	9	93	8.82	91.18
*bla*IMP	100	5	95	5.00	95.00
*bla*ROB	16	1	15	6.25	93.75
*bla*KPC	33	2	31	6.06	93.94
*bla*CTX	31	2	29	6.45	93.55
*bla*CMY	28	5	23	2.87	97.13
*bla*DHA	21	1	20	4.76	95.24
*bla*P	16	1	15	6.25	93.75
*bla*CFX	9	1	8	11.11	88.89
*bla*LAP	10	3	7	30.00	70.00
*bla*LAT	3	0	3	0.00	100.00
*bla*BIL	1	0	1	0.00	100.00

**Table 2:** Primers sequences. Each primer set* was designed to identify all the members of beta-lactamase gene family reported in the GenBank during the 2015 year.

*Gene Family*	*Primer Name*	*Primer Sequence (5′ to 3′)*	*Tm (°C)*	*Position*	*Amplicon (bp)*
*bla*OXA	BlaOXA-FW	GGTTTCGGTAATGCTGAAATTGG	61.18	214–236	114
	BlaOXA-RW	GCTGTGTATGTGCTAATTGGGA	61.19	327–306	
*bla*VIM	BlaVIM-FW	CGACAGTCARCGAAATTCC	61.39	105–123	133
	BlaVIM-RW	CAATGGTCTSATTGTCCGTG	61.34	238–219	
*bla*SHV	BlaSHV-FW1	CGTAGGCATGATAGAAATGGATC	61.04	133–155	106
	BlaSHV-RW1	CGCAGAGCACTACTTTAAAGG	61.33	239–218	
	BlaSHV-FW2	GCCTCATTCAGTTCCGTTTC	61.62	399–418	141
	BlaSHV-RW2	CCATTACCATGAGCGATAACAG	61.22	540–518	
*bla*TEM	BlaTEM-FW	GCCAACTTACTTCTGACAACG	61.80	1699–1719	213
	BlaTEM-RW	CGTTTGGAATGGCTTCATTC	60.13	1912–1892	
*bla*IMP	BlaIMP-FW1	GGAATAGA**R**TGGCTTAAYTCTCG	60.92	319–332	183
	BlaIMP-RW1	C**Y**A**S**TA**S**GTTATCT**K**GAGTGTG	62.45	502–480	
	BlaIMP-FW2	GGTGGAATAGA**R**TGGCTTAA**Y**TC	61.11	316–339	192
	BlaIMP-RW2	CCAAACCACTACGTTATCT**K**GAG	61.29	508–485	
*bla*ROB	BlaROB-FW	CCAACATCGTGGAAAGTGTAG	61.27	718–739	126
	BlaROB-RW	GTAAATTGCGTACTCATGATTGC	60.90	844–821	
*bla*KPC	BlaKPC-FW	GCTAAACTCGAACAGGACTTTG	61.79	100–121	117
	BlaKPC-RW	CTTGAATGAGCTGCACAGTG	61.90	216–197	
*bla*CTX	BlaCTX-FW1	GATACCGCAGATAATACGCAG	60.79	161–181	116
	BlaCTX-RW1	CGTTTTGCGTTTCACTCTG	60.28	276–258	
	BlaCTX-FW2	GCTGATTCTGGTCACTTACTTC	61.02	789–810	83
	BlaCTX-RW2	CGCCGACGCTAATACATC	60.69	855–872	
	BlaCTX-FW3	CTGCTTAACTACAATCCSATTGC	62.17	314–336	226
	BlaCTX-RW3	GGAATGGCGGTATT**K**AGC	60.86	539–522	
*bla*CMY	BlaCMY-FW1	GTTTGAGCTAGGATCGGTTAG	60.25	337–357	123
	BlaCMY-RW1	CTGTTTGCCTGTCAGTTCTG	61.48	460–441	
	BlaCMY-FW2	GAACGAAGGCTACGTAGCT	61.71	213–231	160
	BlaCMY-RW2	CTGAAACGTGATTCGATCATCA	61.08	372–351	
*bla*DHA	BlaDHA-FW1	GCATATTGATCTGCATATCTCCAC	61.60	399–422	200
	BlaDHA-RW1	GCTGCTGTAACTGTTCTGC	61.62	598–580	
	BlaDHA-FW2	GCGGATCTGCTGAATTTCTATC	61.54	464–485	147
	BlaDHA-RW2	GCAGTCAGCAACTGCTCATAC	61.05	610–591	
	BlaDHA-FW3	GTAAGATTCCGCATCAAGCTG	61.74	430–450	117
	BlaDHA-RW3	GGGTTATCTCACACCTTTATTACTG	61.08	546–522	
*bla*P	BlaP-FW	GGAGAATATTGGGATTACAATGGC	61.74	271–294	204
	BlaP-RW	CGCATCATCGAGTGTGATTG	61.80	474–455	
*bla*CFX	BlaCFX-FW	CCAGTCATATCATTGACAGTGAG	60.86	437–459	177
	BlaCFX-RW	GACATTTCCTCTTCCGTATAAGC	61.16	613–591	
*bla*LAP	BlaLAP-FW	AGGGCTTGAACAACTTGAAC	61.07	249–268	126
	BlaLAP-RW	GTAATGGCAGCATTGCATAAC	60.59	374–354	
*bla*BIL	BlaBIL-FW	GCCGATATCGTTAATCGCAC	61.65	100–119	128
	BlaBIL-RW	GTTATTGGCGATATCGGCTTTA	60.98	227–206	

* All primers were designed in PerlPrimer v1.1.21 running under Debian 8 OS.

**Table 3:** Characteristics of whole genome sequencing of the bacteria isolated form of roads cultured from ready-to-eat vegetables.

*Bacteria*	*CDS*	*Number of Sequence Contigs*	*Assembled Genome Size (bp)*	*Reported Genomic Size (bp)*	*Genomic Size Difference*
*Enterobacter cloacae*	4545	1484	4,982,176	4,772,910	209,266
*Serratia marcescens*	6596	8889	5,681,210	5,241,455	439,755
*Klebsiella pneumoniae*	5071	583	5,479,173	5,315,120	164,053
*Serratia fonticola*	5945	916	6,483,043	6,000,511	482,532
*Enterobacter aerogenes*	4545	1484	5,578,724	5,280,350	298,374

**Table 4:** Frequency of beta-lactamase gene families identified by end point PCR in roads cultured from ready-to-eat vegetables.

*Gen Family*	*n*	*Frequency*
*bla*OXA	2	16.7
*bla*VIM	4	33.3
*bla*SHV	11	91.6
*bla*TEM	1	8.3
*bla*IMP	2	16.6
*bla*KPC	1	8.3
*bla*CTX	10	83.3
*bla*DHA	9	75.0
*bla*BIL	11	91.6
*bla*ROB	0	0.0
*bla*CMY	0	0.0
*bla*P	0	0.0
*bla*CFX	0	0.0
*bla*LAP	0	0.0

**Table 5:** Beta-lactamase gen families identified by end point PCR in roads cultured from ready-to-eat vegetables.

*Bacteria*	*Carried Beta-Lactamases Gene Families*									*N*
	*bla*SHV	*bla*CTX	*bla*DHA	*bla*BIL	*bla*KPC	*bla*VIM	*bla*IMP	*bla*OXA	*bla*TEM	
*Klebsiella pneumoniae*	+			+		+				1
*Klebsiella pneumoniae*	+	+	+	+			+			1
*Enterobacter cloacae*	+	+		+						1
*Enterobacter cloacae*		+		+				+	+	1
*Enterobacter cloacae*	+	+	+	+						2
*Enterobacter cloacae*	+	+	+	+			+			1
*Enterobacter cloacae*	+	+	+	+	+					1
*Enterobacter aerogene*	+	+	+	+		+		+		1
*Serratia fonticola*	+		+	+		+				1
*Serratia marcescens*	+	+	+							1
*Serratia marcescens*	+	+	+	+		+				1

**Table 6:** Metagenomic identification of the isolated bacteria form of roads cultured from ready-to-eat vegetables.

*NGS Characteristics*	*Enterobacter Cloacae*	*Serratia Marcescens*	*Klebsiella Pneumoniae*	*Serratia [1 Fonticola]*	*Enterobacter Aerogenes*
Total Reads	4,714,939	241,072	1,523,821	3,848,555	1,331,424
Classified Reads	4,541,943 (96.33%)	183,689 (76.20%)	1,434,672 (94.15%)	3,680,364 (95.63%)	1,200,101 (90.14%)
*Domain*	4,438,740 (94.14%)	182,241 (75.60%)	1,434,058 (94.11%)	3,679,637 (95.61%)	1,198,484 (90.02%)
*Phylum*	4,401,268 (93.35%)	179,018 (74.26%)	1,427,130 (93.65%)	3,678,453 (95.58%)	1,193,110 (89.61%)
*Class*	4,397,652 (93.27%)	177,491 (73.63%)	1,425,587 (93.55%)	3,619,438 (94.05%)	1,190,902 (89.45%)
*Order*	4,394,905 (93.21%)	178,775 (74.16%)	1,419,060 (93.13%)	3,609,215 (93.78%)	1,188,531 (89.27%)
*Family*	4,395,029 (93.21)	178,735 (74.14%)	1,419,175 (93.13%)	3,603,190 (93.62%)	1,188,519 (89.27%)
*Gender*	1,730,480 (36.70%)	176,087 (73.04%)	1,348,898 (88.52%)	3,600,628 (93.56%)	1,141,985 (85.77%)
*Species*	1,685,267 (35.74%)	170,381 (70.68%)	1,219,856 (80.05%)	3,572,513 (92.83%)	1,123,080 (84.35%)

**Table 7:** Genotype of the beta-lactama antibiotic resistome identified by next generation sequencing in roads cultured from ready-to-eat vegetables.

*Bacteria*	*Beta-Lactamases Families*					
	*SHV*	*CTX*	*DHA*	*BIL*	*KPC/VIM/IMP*	*OXA*
*Enterobacter cloacae*	*bla*SHV-12	*bla*CTX-M-15	*bla*DHA-1	*bla*BIL ***	*bla*KPC-2	
*Serratia marcescens*	*bla*SHV-1	*bla*CTX-M-3	*bla*DHA-1	*bla*BIL ***	*bla*VIM-2	
*Klebsiella pneumoniae*	*bla*SHV-12	*bla*CTX-M-15	*bla*DHA-1	*bla*BIL ***	*bla*IMP ***	
*Serratia fonticola*	*bla*SHV-12	*bla*VIM-1	*bla*DHA-1	*bla*BIL ***		
*Enterobacter aerogene*	*bla*SHV-1	*bla*CTX-M-1	*bla*DHA-1	*bla*BIL ***	*bla*VIM-2	*bla*OXA-9

*** No member of this gene family was found in the bioinformatic analysis of WGS.
